# Supplementary material for: Structure of SHOC2-KRAS-PP1C complex reveals RAS isoform-specific determinants and insights into targeting complex assembly by RAS inhibitors
Source: Nat Commun. 2026 Jan 10;17:1614. doi: 10.1038/s41467-026-68319-1 (PMC12905138; doi:10.1038/s41467-026-68319-1)
Supplement: Supplementary file 1 — Supplementary Information [file 41467_2026_68319_MOESM1_ESM.pdf]

Structure of SHOC2-KRAS-PP1C Complex Reveals RAS Isoform-Specific Determinants and Insights into Targeting Complex Assembly by RAS inhibitors

Daniel A. Bonsor[1,*], Lorenzo I. Finci[1,*], Jacob R. Potter[1], Lucy C. Young[2], Vanessa E. Wall[1], Ruby Goldstein de Salazar[2], Katie R. Geis[1], Tyler Stephens[3], Joseph Finney[4], Dwight V. Nissley[1], Frank McCormick[1,2], Dhirendra K. Simanshu[1,#]

Supplementary Infomation

- Supplementary Figures 1-10
- Supplementary Tables 1-3

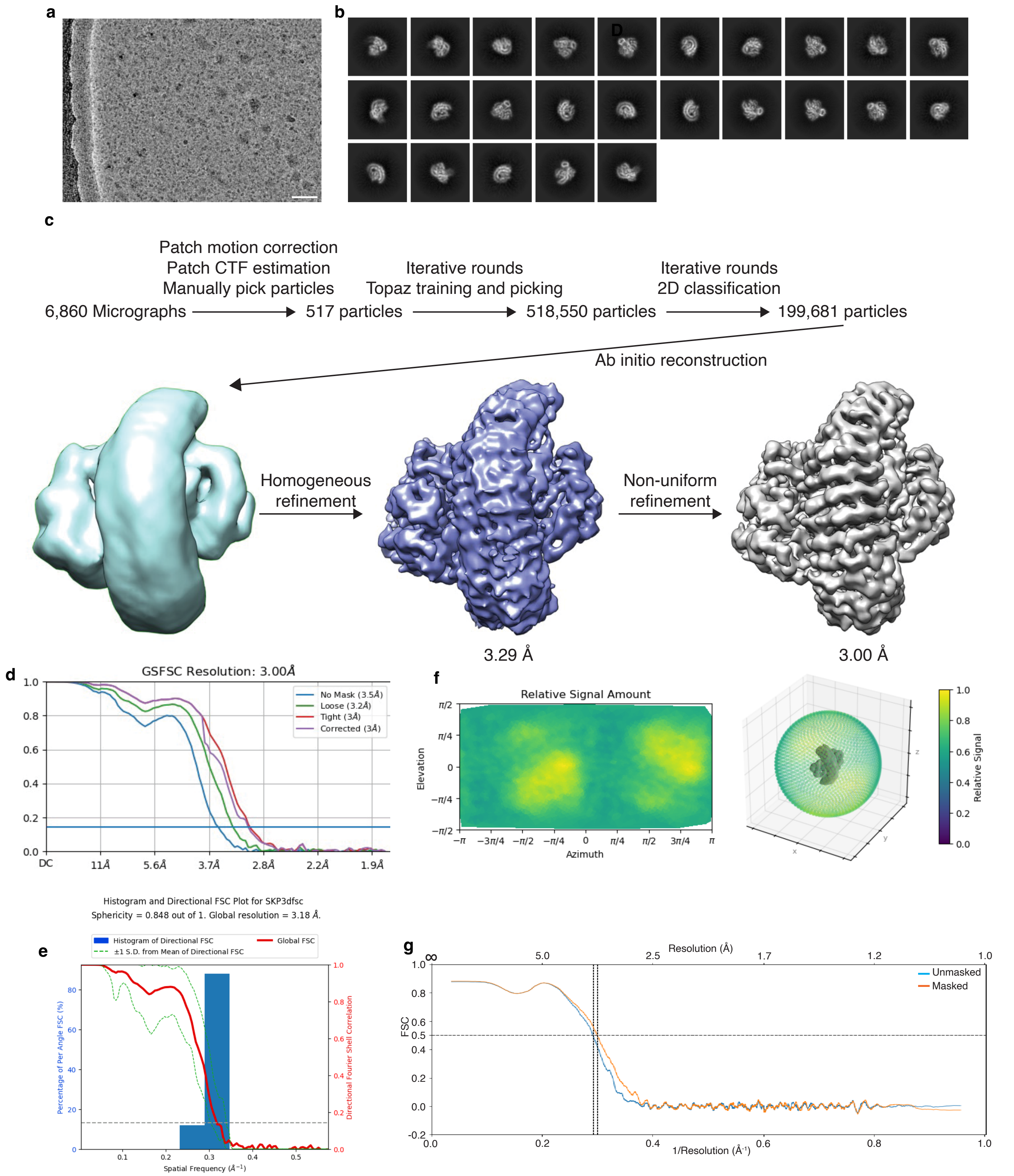

Supplementary Figure 1. Cryo-EM processing workflow for the stabilized SKP complex. (a) Exemplar electron micrograph of vitrified stabilized SKP complex; scale bar, 50 nm. **(b)** Representative 2D class averages of particles selected from the micrograph. **(c)** Schematic overview of the cryo-EM data processing pipeline performed in cryoSPARC. See Materials and Methods for details. **(d)** Gold-standard Fourier Shell Correlation (FSC) curve for the final 3D reconstruction of the complex. **(e)** Histogram and directional FSC Plot that represents a measure of directional resolution anisotropy computed on the 3DFSC webserver. **(f)** The relative signal visualized in a 2D azimuth-elevation chart (left), and in a 3D colored scatter plot (right) calculated in cryoSPARC. Low relative signal represented by darker colors correspond to under-represented views. **(g)** The FSC model-map that shows the Fourier shell coefficient curve base on the model map with and without masking. The intersection of the curves with FSC = 0.5 are depicted.

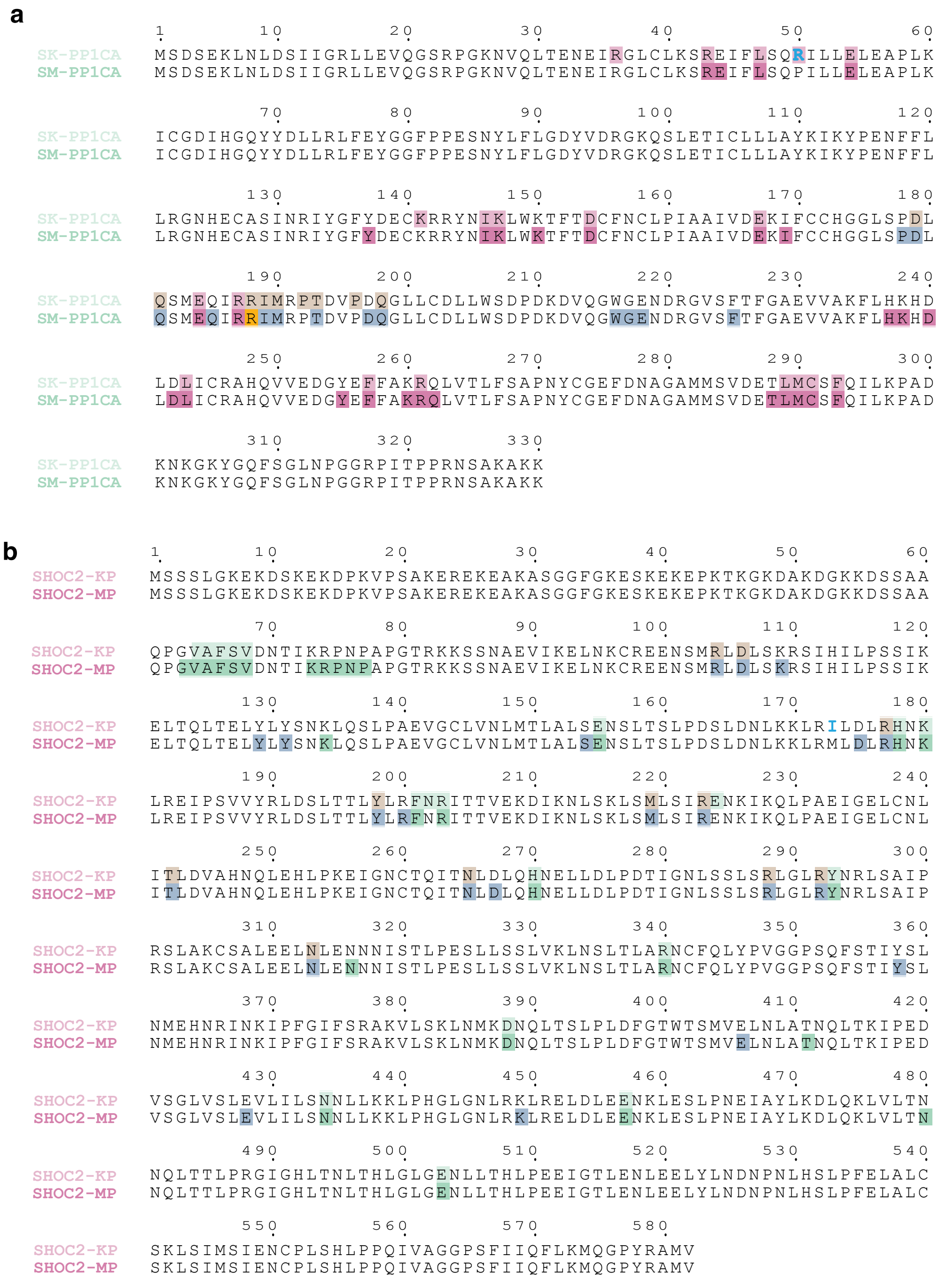

Supplementary Figure 2. Interactions of PP1CA and SHOC2 with KRAS and MRAS. Sequence alignment of **(a)** PP1CA and **(b)** SHOC2 from the stabilized SKP, and SMP complexes, highlighting residues that interact with SHOC2 (pink), PP1CA (green), KRAS (light brown), and MRAS (blue). PP1CA-R188 from the SMP complex is shown in orange, as it contacts both SHOC2 and MRAS. Mutations introduced in the stabilized SKP complex—PP1CA-P50R and SHOC2-M173I—are indicated as bold blue residues.

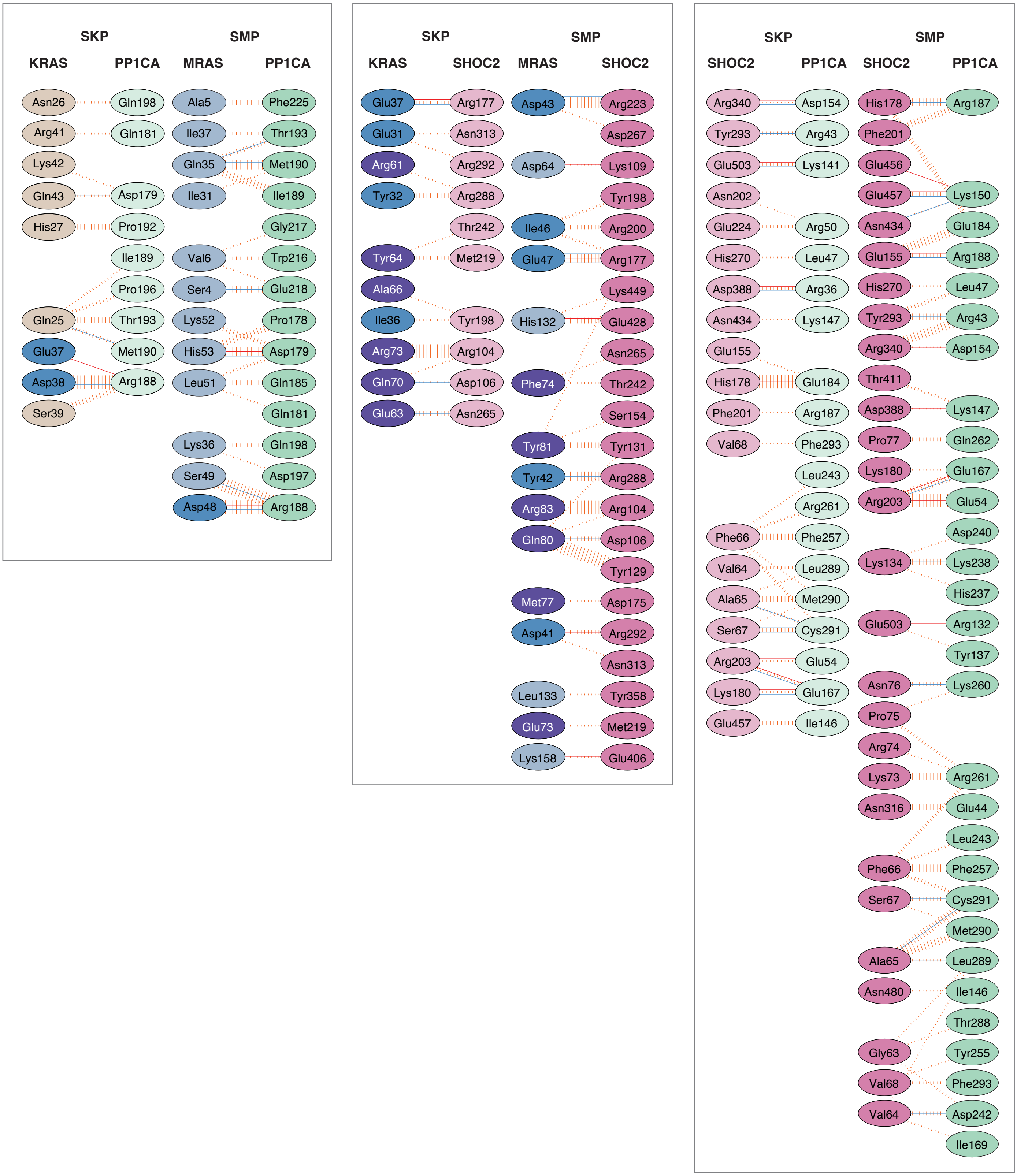

Supplementary Figure 3. Comparison of the interaction networks in the stabilized SKP, and SMP complexes. Schematic representation of the RAS-PP1CA, RAS-SHOC2 and SHOC2-PP1CA interactions as analyzed by PDBSum. The interactions are colored using the following notations: hydrogen bonds as solid blue lines and non-bonded contacts as dashed orange lines (the width of the lines is proportional to the number of atomic contacts). SHOC2 and PP1C residues are shown pinks and greens. KRAS and MRAS residues are shown in light brown and blue, respectively. Switch-I and -II of KRAS and MRAS are shown in dark blue and purple,respectively.

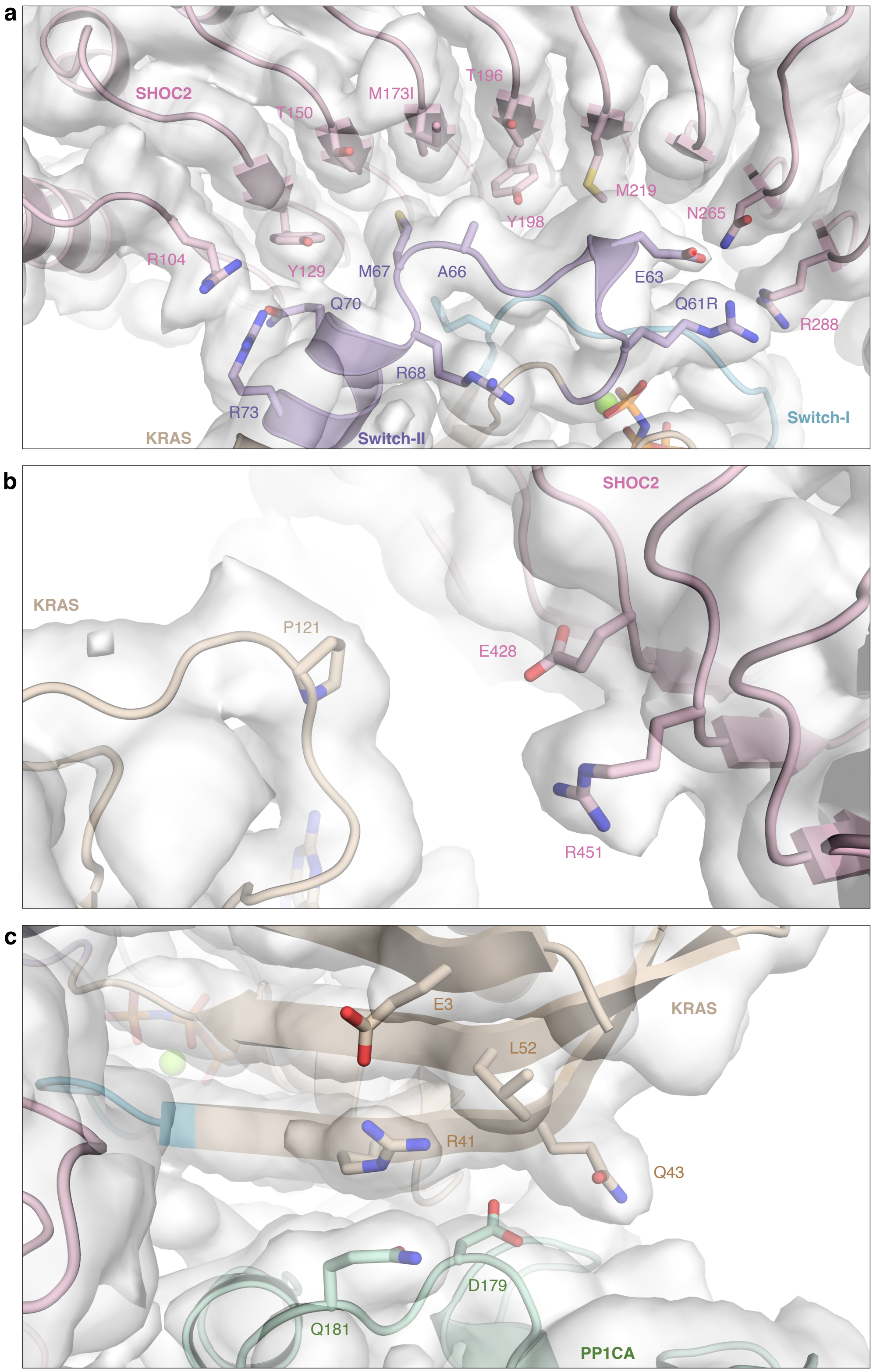

Supplementary Figure 4. CryoEM map associated with the interfaces of the stabilized SKP complex. The 3Å resolution DeepEMhancer sharpened map highlighting key interactions; **(a)** KRAS Switch interactions with SHOC2; **(b)** lack of KRAS C-terminal with SHOC2 and **(c)** KRAS interswitch interactions with PP1CA.

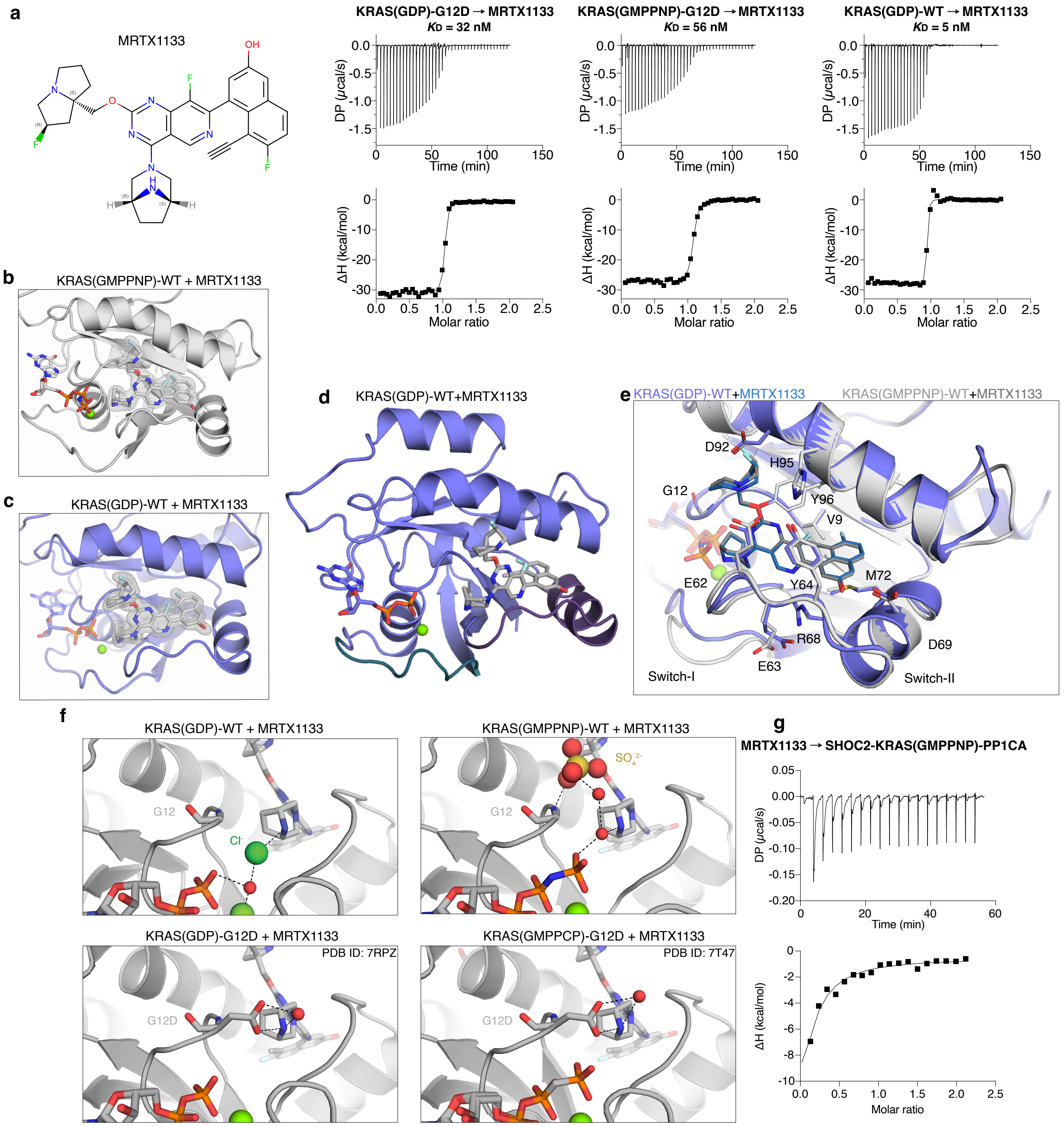

Supplementary Figure 5. Biophysical and structural analysis of MRTX1133 binding to KRAS. (a) ITC traces showing MRTX1133 (skeletal formula) binding to KRAS(GDP)-G12D, KRAS(GMPPNP)-G12D, and KRAS(GDP)-WT. The K_D is calculated from two technical replicates. Omit maps contoured at 1.5σ showing electron density for MRTX1133 bound to **(b)** KRAS(GMPPNP)-WT and **(c)** KRAS(GDP)-WT. **(d)** Crystal structure of the KRAS(GDP)-WT–MRTX1133 complex, with KRAS in blue and MRTX1133 in gray. **(e)** Structural superposition of KRAS(GMPPNP)-WT–MRTX1133 and KRAS(GDP)-WT–MRTX1133 complexes. **(f)** Close-up view of the interaction between the bridged piperazine moiety of MRTX1133 and the residue at the 12th position in KRAS-WT and KRAS-G12D. **(g)** ITC trace showing that MRTX1133 weakly disrupts the SKP complex.

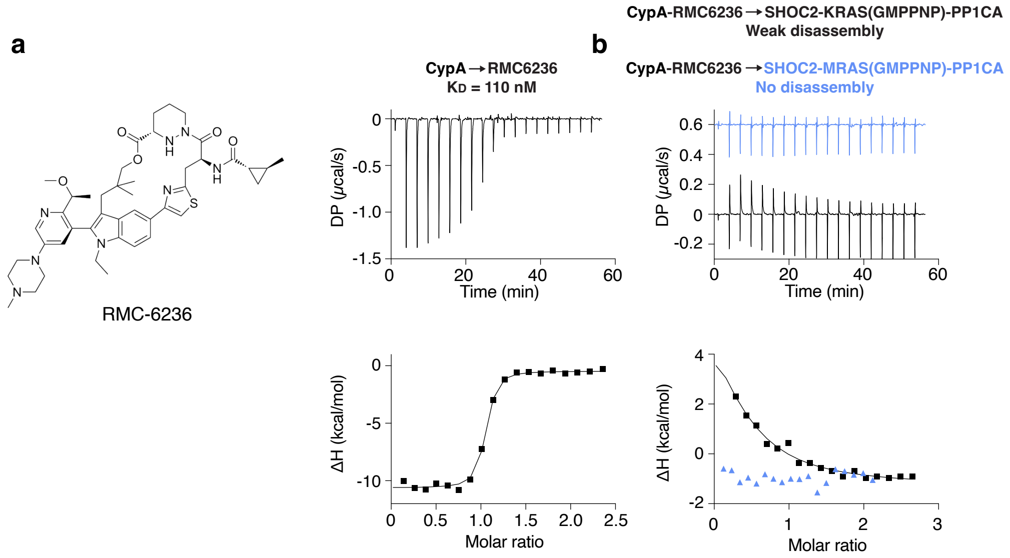

Supplementary Figure 6. Differential impact of RMC-6236–CypA on KRAS and MRAS complexes. (a) ITC trace showing binding of RMC-6236 (skeletal formula) to CypA. The K_D is calculated from two technical replicates. **(b)** ITC profiles showing the RMC6236-CypA complex weakly disrupts the SKP complex (black) but does not disassemble the SMP complex (blue).

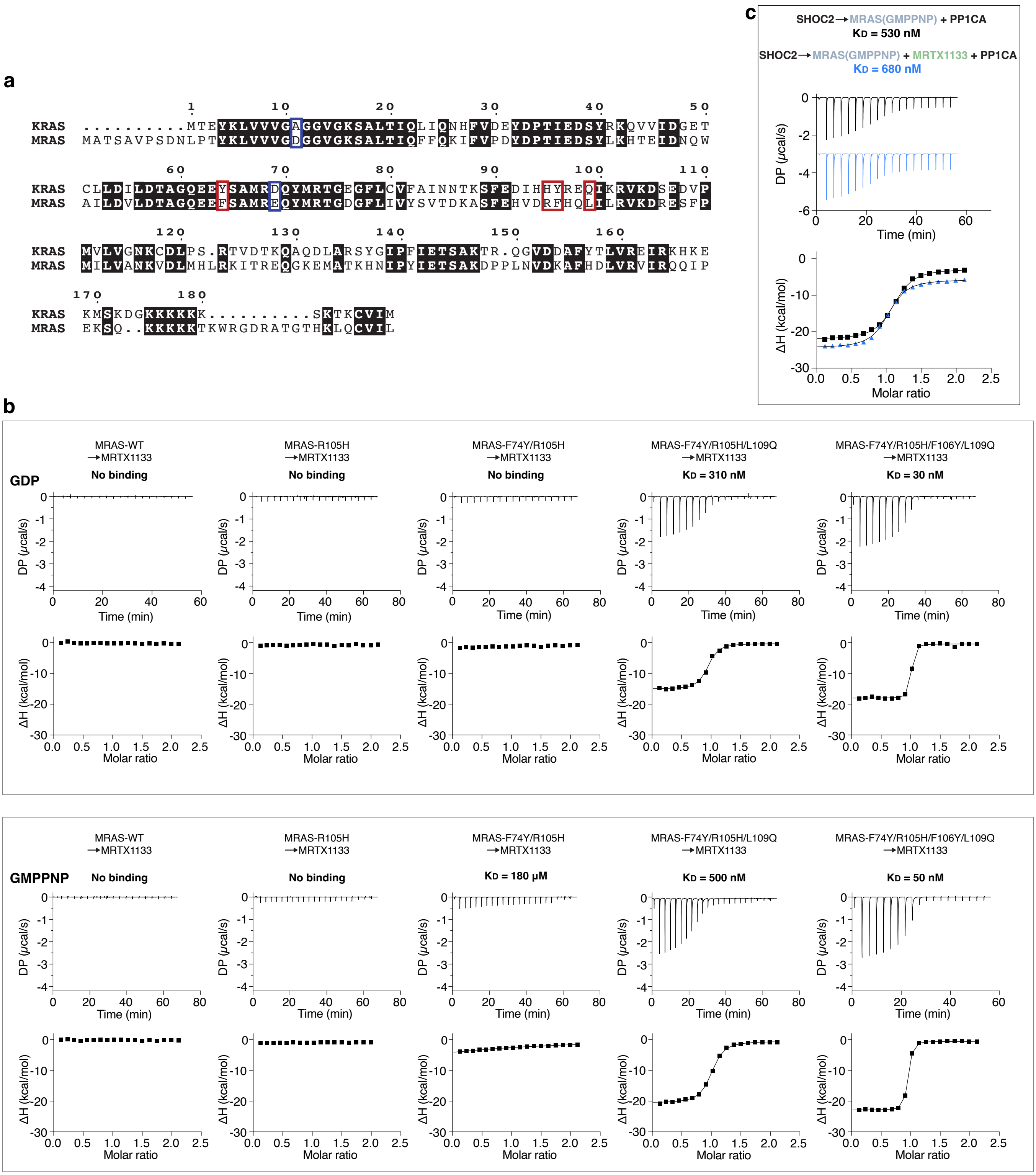

Supplementary Figure 7. Engineering MRTX1133 sensitivity into MRAS and its impact on SMP assembly. (a) Sequence alignment of KRAS and MRAS highlighting differences within the MRTX1133 binding pocket. Mutated MRAS residues are boxed in red; conserved residues D21 and E79, which contribute to the binding pocket but were not mutated, are boxed in blue. **(b)** ITC traces of MRAS-WT, MRAS-R105H, MRAS-F74Y/R105H, MRAS-F74Y/R105H/L109Q and MRAS F74Y/R105H/F106Y/L109Q binding to MRTX1133 in the GDP- and GMPPNP-bound states.The K_D is calculated from two technical replicates. **(c)** SMP complex formation in the absence (black) and presence (blue) of MRTX1133, showing that the engineered MRAS variant still assembles the SMP complex but is inhibited by the compound.

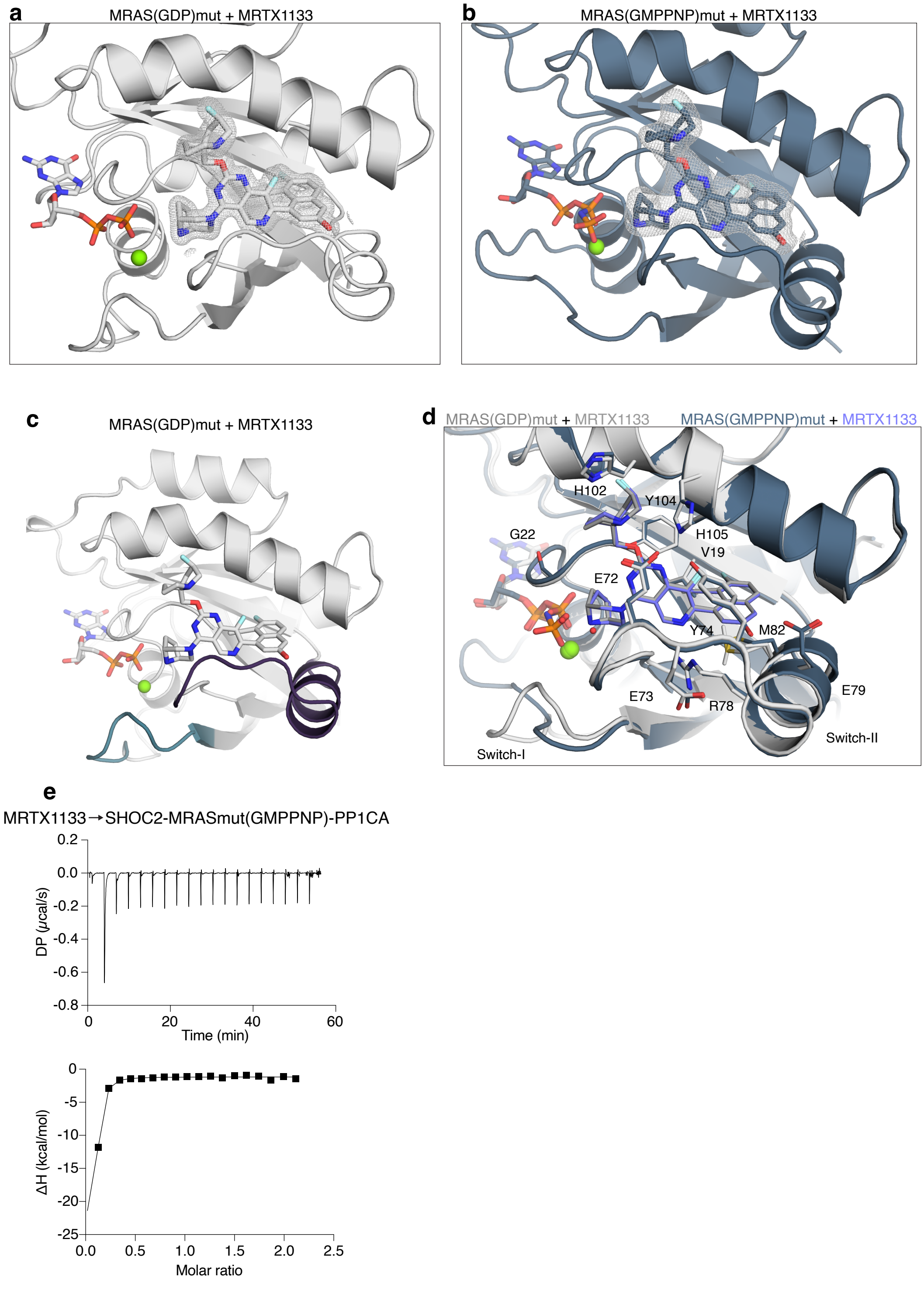

Supplementary Figure 8. Biophysical and structural characterization of MRTX1133 binding to mutated MRAS. Omit map showing electron density corresponding to MRTX1133 contoured at 1.5 sigma bound to **(a)** MRASmut(GDP) and **(b)** MRASmut(GMPPNP). **(c)** Crystal structure of MRASmut(GDP). **(d)** Superposition of MRASmut(GMPPNP)-MRTX1133 and MRASmut(GDP)-MRTX1133 structures. **(e)** ITC trace demonstrating that MRTX1133 is not capable of disrupting the SMmutP complex. The excess heat in the first injection is due to a slight excess of MRASmut binding to MRTX1133.

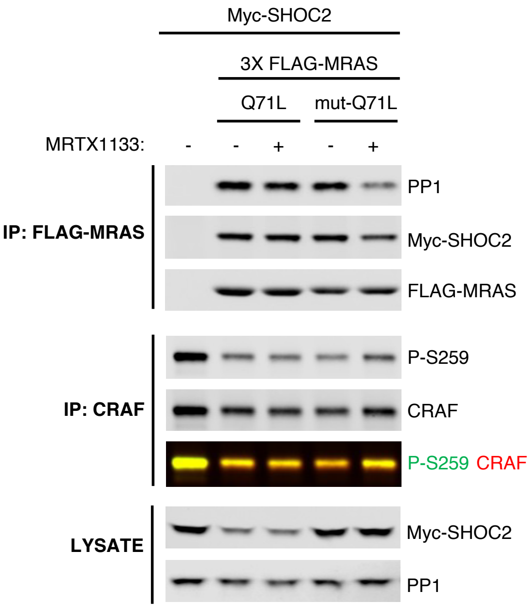

Supplementary Figure 9. MRTX1133 impairs MRASmut-Q71L complex formation and dephosphorylation of RAF in quadruple knockout (H/N/K/MRAS) 293 cells. 3X FLAG-MRAS-Q71L and MRASmut-Q71L immunoprecipitates were probed for Myc-SHOC2 and PP1 following 3 μM MRTX1133 treatment for 4 hours. Endogenous CRAF immunoprecipitates from the same cells were probed for P-S259-CRAF. Full uncropped scans of blot images are provided in the Source Data.

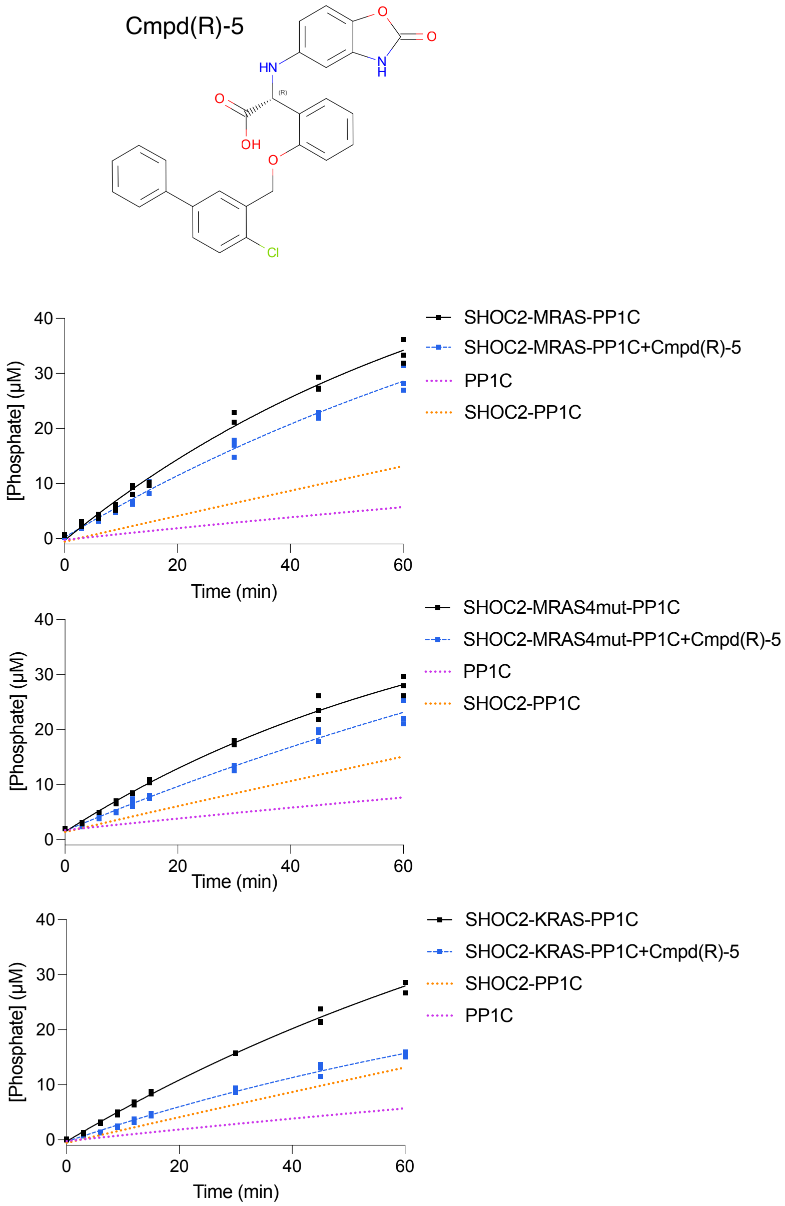

Supplementary Figure 10. Inhibition of SHOC2-RAS-PP1C complexes by Novartis Cmpd(R)-5. Phosphate production by the SHOC2-MRAS-PP1C, SHOC2-MRASmut-PP1C and SHOC2-KRAS-PP1C complexes, respectively, from a CRAF CR2-pS peptide as a substrate in the presence or absence of Cmpd(R)-5 (skeletal formula). n=3. Magenta and orange dashed lines, represent the basal activity of PP1C and PP1C-SHOC2 from Fig. 7f.

Supplementary Table 1: Summary of measured protein-protein and protein-small molecule interactions.

Interaction	K_D (nM)*	ΔH (kcal mol^{-1})*	-TΔS (kcal mol^{-1})*	Technique	Ref
SHOC2→MRAS+PP1CA	922±189	-3.9±0.5	5.6±0.5	ITC	This study
	200			BLI	14
	31.3			SPR	15
	115			SPR	16
	120			SPR	13
SHOC2→KRAS+PP1CA	6050±1990	-9.0±1.0	1.9±1.2	ITC	This study
	425			SPR	15
	700			SPR	13
SHOC2→HRAS+PP1CA	2000			SPR	13
SHOC2→NRAS+PP1CA	4000			SPR	13
	679			SPR	15
SHOC2(M173L)→KRAS(Q61R)+PP1CA(P50R)	154±1	-1.0±0.6	1.7±0.5	ITC	This study
KRAS(GDP)-WT→MRTX1133	5±1	-8.1±0.1	16.5±0.2	ITC	This study
	0.787			SPR	49
KRAS(GMPPNP)-WT→MRTX1133	15.4±4	-7.6±0.1	16.9±0.1	ITC	This study
	15.6			SPR	49
KRAS(GDP)-G12D→MRTX1133	32±5	-0.5±0.2	20.2±0.1	ITC	This study
	0.0002			SPR	29
KRAS(GMPPNP)-G12D→MRTX1133	56±11	-8.9±1.5	19.0±1.6	ITC	This study
	0.140			SPR	30
SHOC2→KRAS+PP1CA (5% DMSO)	7900±540	-7.0±0.5	10.1±0.5	ITC	This study
SHOC2→KRAS-MRTX11+PP1CA (5% DMSO)	No complex assembly			ITC	This study
CypA→RMC6236	108±22	-0.0±0.1	0.5±0.2	ITC	This study
	55.3			SPR	28
KRAS→CypA-RMC6236	127±17	-6.1±0.1	-3.3±0.1	ITC	This study
	154			BLI	28
MRAS→CypA-RMC6236	No complex assembly			ITC	This study
CypA-RMC6236→SHOC2-KRAS-PP1CA	34600±1300[#]			ITC	This study
CypA-RMC6236→SHOC2-MRAS-PP1CA	401±82	-1.6±0.3	12.9±0.5	ITC	This study
MRAS(GDP)-WT→MRTX1133	No binding			ITC	This study
MRAS(GMPPNP)-WT→MRTX1133	No binding			ITC	This study
MRAS(GDP)-R105H→MRTX1133	No binding			ITC	This study
MRAS(GMPPNP)-R105H→MRTX1133	No binding			ITC	This study
MRAS(GDP)-F74Y/R105H→MRTX1133	No binding			ITC	This study
MRAS(GMPPNP)-F74Y/R105H→MRTX1133	183000±32500[#]			ITC	This study
MRAS(GDP)-F74Y/R105H/L109Q→MRTX1133	310±61	-14.5±0.4	5.6±0.5	ITC	This study
MRAS(GMPPNP)-F74Y/R105H/L109Q →MRTX1133	496±2	-20.0±0.1	11.4±0.1	ITC	This study
MRAS(GDP)-F74Y/R105H/F106Y/L109Q →MRTX1133	28±0	-17.8±0.2	7.5±0.2	ITC	This study
MRAS(GMPPNP)-F74Y/R105H/F106Y/L109Q →MRTX1133	53±11	-22.9±0.6	13.0±0.7	ITC	This study
SHOC2 →MRAS-F74Y/R105H/F106Y/L109Q+PP1CA (5% DMSO)	1385±85	-15.7±0.4	7.7±0.4	ITC	This study
SHOC2 →MRAS-F74Y/R105H/F106Y/L109Q-MRTX1133+PP1CA (5% DMSO)	No complex assembly			ITC	This study

*Average of duplicate technical runs with the range of values shown.
[#]Binding is too weak to accurately determine ΔH and -TΔS.

Supplementary Table 2: Cryo-EM data collection, refinement and validation statistics.

	**SKP: SHOC2(M173I)-KRAS(Q61R)-PP1CA(P50R)** (EMD-70159) (PDB 9O65)
**Data collection and processing**	
Electron Microscope	NCEF Titan Krios
Detector	Gatan K3
Magnification	105,000
Voltage (kV)	300
Electron exposure (e–/Å^2)	52.3
Defocus range (μm)	-0.75 to -2.25
Pixel size (Å)	0.873
Symmetry imposed	C1
Initial particle count (no.)	518,550
Final particle count (no.)	199,681
Map resolution (Å)	3.0
FSC threshold	0.143
Map resolution range (Å)	2.91 – 11.62
**Refinement**	
Initial models used (PDB code)	7TVF, 5UFE
Model resolution (Å) @ FSC threshold	3.3 @ FSC=0.5
Map sharpening method	CryoSPARC B factor sharpening (-80)
Correlation Coefficient (CCmask)	0.81
Model composition	
Non-hydrogen atoms	7,348
Protein residues	918
Ligands	1:GNP, 1:MG, 2:MN
B factors (Å^2) (min/max/mean)	
Protein	52.60/229.01/96.12
Ligand	77.02/95.40/87.26
R.m.s. deviations	
Bond lengths (Å)	0.006
Bond angles (°)	1.206
Validation	
MolProbity score	1.54
Clashscore	5.62
Rotamer outliers (%)	0.60
CaBLAM outliers (%)	1.00
Ramachandran plot	
Favored (%)	96.48
Allowed (%)	3.52
Outliers (%)	0

Supplementary Table 3: X-ray crystallographic data collection and refinement statistics.

	**KRAS(GDP) + MRTX1133**	**KRAS(GMPPNP) + MRTX1133**	**MRASmut(GDP) + MRTX1133**	**MRASmut(GMPPNP) + MRTX1133**
**PDB ID**	9O0N	9O0O	9O0P	9O0Q
**Data collection**				
Space group	$P\ 2_1\ 2_1\ 2_1$	P 2 3	$P\ 2_1\ 2_1\ 2_1$	I 2 2 2
Cell dimensions				
a, *b*, *c* (Å)	39.79, 50.80, 90.87	89.43, 89.43, 89.43	40.00, 62.61, 140.86	82.06, 110.88, 194.08
α, β, γ (°)	90, 90, 90	90, 90, 90	90, 90, 90	90, 90, 90
Resolution (Å)	50.80-1.40 (1.42-1.40)*	89.43-1.90 (1.94-1.90)	62.61-1.50 (1.53-1.50)	48.52-1.90 (1.94-1.90)
Unique reflections	37144 (1824)	19081 (1239)	57604 (2778)	69697 (4414)
R_{merge}	0.082 (1.29)	0.101 (1.50)	0.085 (1.68)	0.063 (1.92)
R_{pim}	0.039 (0.69)	0.062 (0.92)	0.034 (0.69)	0.026 (0.76)
$I/\sigma I$	13.9 (1.6)	10.2 (1.2)	15.5 (0.9)	23.5 (1.7)
Completeness (%)	99.9 (99.9)	99.9 (100.0)	99.8 (98.4)	99.7 (99.1)
Redundancy	9.5 (8.5)	6.7 (7.0)	13.4 (13.3)	13.7 (14.2)
Wilson B-factor (Å^2)	19	31	20	36
CC(1/2)	0.998 (0.61)	0.997 (0.45)	0.999 (0.67)	1.000 (0.80)
**Refinement**				
Resolution (Å)	45.43-1.40	89.43-1.90	57.21-1.50	44.70-1.90
No. reflections	37040 (1882)#	18175 (891)	57502 (2899)	69597 (3557)
R_{work} / R_{free}	16.02/19.75	16.93/20.70	19.46/22.14	17.68/20.71
No. atoms	1627	1637	3304	4877
Protein	1391	1345	2791	4079
Ligand/ion	83	107	146	312
Water	153	185	368	486
B-factors (Å^2)	29	40	33	57
RAS proteins	26	39	33	58
Ligand/ion	21	38	36	58
Water	40	47	39	55
R.m.s. deviations				
Bond lengths (Å)	0.012	0.008	0.013	0.016
Bond angles (°)	1.280	0.929	1.412	1.509
Ramachandran plot				
Favored (%)	98.2	98.2	98.8	97.6
Allowed (%)	1.8	1.8	1.2	2.4
Outliers (%)	0	0	0	0

* Values in parentheses refer to the highest resolution shell.

# Values in parentheses refer to the number of R_{free} reflections.
