# Supplementary material for: Structure of SHOC2-KRAS-PP1C complex reveals RAS isoform-specific determinants and insights into targeting complex assembly by RAS inhibitors
Source: Nat Commun. 2026 Jan 10;17:1614. doi: 10.1038/s41467-026-68319-1 (PMC12905138; doi:10.1038/s41467-026-68319-1)
Supplement: Supplementary file 2 — Reporting Summary [file 41467_2026_68319_MOESM2_ESM.pdf]

nature portfolio

Corresponding author(s): Dhirendra Simanshu

Last updated by author(s): Dec 2, 2025

Reporting Summary

Nature Portfolio wishes to improve the reproducibility of the work that we publish. This form provides structure for consistency and transparency in reporting. For further information on Nature Portfolio policies, see our Editorial Policies and the Editorial Policy Checklist.

Statistics

For all statistical analyses, confirm that the following items are present in the figure legend, table legend, main text, or Methods section.

n/a	Confirmed	
☐	☒	The exact sample size (*n*) for each experimental group/condition, given as a discrete number and unit of measurement
☐	☒	A statement on whether measurements were taken from distinct samples or whether the same sample was measured repeatedly
☒	☐	The statistical test(s) used AND whether they are one- or two-sided<br>*Only common tests should be described solely by name; describe more complex techniques in the Methods section.*
☒	☐	A description of all covariates tested
☒	☐	A description of any assumptions or corrections, such as tests of normality and adjustment for multiple comparisons
☐	☒	A full description of the statistical parameters including central tendency (e.g. means) or other basic estimates (e.g. regression coefficient) AND variation (e.g. standard deviation) or associated estimates of uncertainty (e.g. confidence intervals)
☒	☐	For null hypothesis testing, the test statistic (e.g. *F*, *t*, *r*) with confidence intervals, effect sizes, degrees of freedom and *P* value noted<br>*Give P values as exact values whenever suitable.*
☒	☐	For Bayesian analysis, information on the choice of priors and Markov chain Monte Carlo settings
☒	☐	For hierarchical and complex designs, identification of the appropriate level for tests and full reporting of outcomes
☒	☐	Estimates of effect sizes (e.g. Cohen's *d*, Pearson's *r*), indicating how they were calculated

Our web collection on statistics for biologists contains articles on many of the points above.

Software and code

Policy information about availability of computer code

Data collection	*Provide a description of all commercial, open source and custom code used to collect the data in this study, specifying the version used OR state that no software was used.*
Data analysis	xds (2023)<br>CCP4 (v9.0)<br>Phenix (v1.21)<br>PyMol (v3.0)<br>Coot (v0.95)<br>Prism10<br>MicroCal PEAQ-ITC analysis software (v1.41)<br>ISOLDE (v1.4)<br>IMODFIT (v1.03)<br>DeepEMhancer (v0.13)<br>CryoSPARC (v4.0)

For manuscripts utilizing custom algorithms or software that are central to the research but not yet described in published literature, software must be made available to editors and reviewers. We strongly encourage code deposition in a community repository (e.g. GitHub). See the Nature Portfolio guidelines for submitting code & software for further information.

Data

Policy information about availability of data

All manuscripts must include a data availability statement. This statement should provide the following information, where applicable:

- Accession codes, unique identifiers, or web links for publicly available datasets
- A description of any restrictions on data availability
- For clinical datasets or third party data, please ensure that the statement adheres to our policy

The atomic coordinates and structure factors have been deposited in the Protein Data Bank and can be accessed using accession numbers 9O65/EMD-70159 [http://doi.org/10.2210/pdb9O65/pdb] (stabilized SKP complex), 9O0N [http://doi.org/10.2210/pdb9O0N/pdb] (KRAS(1-169)GDP with MRTX1133), 9O0O [http://doi.org/10.2210/pdb9O0O/pdb] (KRAS (1-169)GMPPNP with MRTX1133), 9O0P [http://doi.org/10.2210/pdb9O0P/pdb] (MRASmut(1-178)GDP with MRTX1133) and 9O0Q [http://doi.org/10.2210/pdb9O0Q/pdb] (MRASmut(1-178)GMPPNP with MRTX1133). The structures used as initial models for molecular replacement are available in the PDB under accession codes 1X1R [http://doi.org/10.2210/pdb1X1R/pdb] (MRAS), 7RPZ [http://doi.org/10.2210/pdb7RPZ/pdb] (KRAS(G12D)GDP-MRTX1133), and 7TVF [http://doi.org/10.2210/pdb7TVF/pdb] (SMP complex). Structures utilized for superpositions, and analysis can be found in the PDB using accession codes 1NVU [http://doi.org/10.2210/pdb1NVU/pdb] (HRAS-SOS1), 6OB2 [http://doi.org/10.2210/pdb6OB2/pdb] (KRAS+NF1), 6XI7 [http://doi.org/10.2210/pdb6XI7/pdb] (KRAS-CRAF), 7LC1 [http://doi.org/10.2210/pdb7LC1/pdb] (KRAS-Sin1), 7RPZ [http://doi.org/10.2210/pdb7RPZ/pdb] (KRAS(G12D)GDP-MRTX1133), 7T47 [http://doi.org/10.2210/pdb7T47/pdb] (KRAS(G12D)GMPPCP-MRTX1133), 7TVF [http://doi.org/10.2210/pdb7TVF/pdb] (SMP complex), 8B69 [http://doi.org/10.2210/pdb8B69/pdb] (KRAS-Rgl2), 9AX6 [http://doi.org/10.2210/pdb9AX6/pdb] (KRAS-RMC6236-CypA) and 9C15 [http://doi.org/10.2210/pdb9C15/pdb] (KRAS-PI3K). The source data underlying Figure 7f-i, and Supplementary Figures 9 and 10 are provided as a Source Data file.

Research involving human participants, their data, or biological material

Policy information about studies with human participants or human data. See also policy information about sex, gender (identity/presentation), and sexual orientation and race, ethnicity and racism.

Reporting on sex and gender	N/A
Reporting on race, ethnicity, or other socially relevant groupings	N/A
Population characteristics	N/A
Recruitment	N/A
Ethics oversight	N/A

Note that full information on the approval of the study protocol must also be provided in the manuscript.

Field-specific reporting

Please select the one below that is the best fit for your research. If you are not sure, read the appropriate sections before making your selection.

☒ Life sciences ☐ Behavioural & social sciences ☐ Ecological, evolutionary & environmental sciences

For a reference copy of the document with all sections, see nature.com/documents/nr-reporting-summary-flat.pdf

Life sciences study design

All studies must disclose on these points even when the disclosure is negative.

Sample size	N/A
Data exclusions	No data were excluded.
Replication	Replicate measurements were taken for ITC and dephosphorylation assay measurements as indicated in methods section of the manuscript.
Randomization	N/A
Blinding	No blinding experiments were performed. An individual or group of people conducted each experiment according to their expertise.

Reporting for specific materials, systems and methods

We require information from authors about some types of materials, experimental systems and methods used in many studies. Here, indicate whether each material, system or method listed is relevant to your study. If you are not sure if a list item applies to your research, read the appropriate section before selecting a response.

Materials & experimental systems

n/a	Involved in the study	
☐	☒	Antibodies
☐	☒	Eukaryotic cell lines
☒	☐	Palaeontology and archaeology
☒	☐	Animals and other organisms
☒	☐	Clinical data
☒	☐	Dual use research of concern
☒	☐	Plants

Methods

n/a	Involved in the study	
☒	☐	ChIP-seq
☒	☐	Flow cytometry
☒	☐	MRI-based neuroimaging

Antibodies

Antibodies used

Myc-tag Rabbit polyclonal Ab (Cell Signaling Technology Cat.# 2272, 1:2000 dilution)
PP1 alpha Rabbit polyclonal Ab (Upstate Cat.# 06-221, 1:2000 dilution)
FLAG Rabbit polyclonal Ab (Sigma-Aldrich Cat.# F7425, 1:4000 dilution)
CRAF Mouse mAb (BD Biosciences Cat.# 610152, 1:2000 dilution)
Anti-Rabbit IgG (H&L) Antibody DyLight 800 (Invitrogen Cat.# SA5-35571, 1:15000 dilution)
Anti-Mouse IgG (light chain) antibody Alexa Fluor 680 (Jackson ImmunoResearch Cat.# 115-625-174, 1:15000 dilution)

Validation

All antibodies were validated by their manufacturers for the application they were used for.

Eukaryotic cell lines

Policy information about cell lines and Sex and Gender in Research

Cell line source(s)

Tni-FNL cells, RASless cells

Authentication

Tni-FNL cells - Genome Assembly and Annotation of the Trichoplusia ni Tni-FNL Insect Cell Line Enabled by Long-Read Technologies (https://doi.org/10.3390/genes10020079. This has been referenced in the paper.)

Triple knockout HRAS/KRAS/NRAS 293 cell line were authenticated in Cuevas-Navarro et al (DOI: 10.1126/sciadv.adf4766).

Generation of quadruple knockout HRAS/NRAS/KRAS/MRAS in this study were confirmed by Western blot and sequencing of the sgRNA target region (see Methods).

Mycoplasma contamination

Tni-FNL cells have been verified as mycoplasma free by the Frederick National Lab Cell Line Development Group.

RASless cell lines were regularly tested and verified to be mycoplasma negative using the MycoAlert PLUS Mycoplasma Detection Kit by Lonza

Commonly misidentified lines
(See ICLAC register)

None

Plants

Seed stocks

N/A

Novel plant genotypes

N/A

Authentication

N/A
